# How Consumer Expertise Influences Preference for Customized Food

**DOI:** 10.3390/foods11162459

**Published:** 2022-08-15

**Authors:** Han Li, Fen Liao, Ping Qing

**Affiliations:** 1 College of Economics and Management, Huazhong Agricultural University, Wuhan 430070, China; 2 School of Insurance, Shandong University of Finance and Economics, Jinan 250014, China

**Keywords:** customization, customized foods, consumer expertise, taste perception, purchase intention

## Abstract

The strategy of food customization has increasingly aroused widespread interest among marketing managers and scholars, but most of them focus on the direct effect of customization on purchase intention. According to the research on self-image-consistent product perceptions, customization may also have an indirect amplification effect. Current research finds food customization will prompt individuals to incorporate their expertise in their perceptions of focal product attributes (taste perception). The findings of two studies demonstrate that food customization and consumer expertise have an interaction effect on consumers’ purchase intention. Specifically, consumers with higher (lower) expertise prefer customized food (standard food). Consumers’ taste perception mediates the interaction effect between food customization and consumer expertise on purchase intention. Finally, these findings provide guidance for marketing managers to adopt customized strategies.

## 1. Introduction

Emulating the application of customization in industrial products, food retailers have increasingly realized the importance of food customization and offer consumers a customization experience. Nestlé, for example, has offered consumers a personalized light meal plan by letting them choose between seven energetic ingredients and three basic ingredients to create their own drinks. Similarly, Subway allows consumers to create custom-made sandwiches by giving customers the opportunity to pick their own bread, meat, side dishes and other ingredients, and the Australian chain salad brand Saladworks invites consumers to create unique salads. According to the Global Dining Out Market 2020–2024, the rise of customized foods will gradually become a major driver of the dining out market, although the scale of the dining out market may drop to $750 billion.

Previous research has shown that customization may have multiple benefits by allowing consumers to select a set of relatively satisfying attribute options [1], giving them greater subjective value [2] and conveying their identity signals [3]. Consumers also show a great feeling of engagement and accomplishment [4,5] and self-integration with the product [6]. Therefore, food customization may increase consumer satisfaction and purchase intention to some extent [7,8]. However, other scholars believe that food customization may bring some negative effects, such as increasing process complexity [9] and decision difficulty [8] or eroding the product’s expertise signaling value [10].

However, except for the above positive or negative direct effect, customization seems to have an indirect amplification effect. Klesse et al. finds that customization can lead consumers to self-image-consistent product perceptions, which means that customized products can be viewed as an extension of a consumer’s self-image [11]. From this perspective, whether consumers prefer customized food may depend on their own self-perception. We presume that consumers who participate in food customization will also perceive the focal food attribute in line with their own characteristics. Importantly, taste perception is a key factor for consumers to evaluate or purchase food [12,13]. Therefore, our work believes that consumers’ evaluation of or preference for customized foods depends on their own food-related expertise. Specifically, consumers with higher (lower) expertise prefer customized food (standard food). Thus, the current research intends to investigate the interaction effect of food customization and consumer expertise on their purchase intention and the mechanism of this effect.

We test our predictions using two food categories (sandwiches and yogurt) in two experiments. We consistently demonstrate that consumers’ preference for customized foods depends on their food-related expertise and consumers’ taste perception mediates the interaction effect between food customization and expertise on purchase intention. Our work makes a number of theoretical contributions to the literature on food customization by demonstrating that consumers do not always prefer customized foods. Furthermore, our work extends self-image-consistent product perceptions to the field of food customization and then reveals the underlying psychological mechanisms in taste perception. This finding also has some important practical implications for targeting and pushing customized foods for consumers with high expertise or similar food consumption experience but not for those with low expertise. On the contrary, for those with low expertise, managers should avoid food customization as far as possible, thereby weakening the adverse effect of customized foods with low perceived taste.

## 2. Conceptual Framework and Hypotheses

### 2.1. Preference for Customized Foods

With the advancement of technology and improvement of service, as well as merchants’ increasing attention to the personalized needs of consumers, many companies adopt customization as an integral part of their business strategy.

With the rise of customization strategies, product customization has also aroused widespread interest among scholars. A large body of consumer research has revealed the reasons for the success of the customization strategy. Previous research shows that customized foods, compared with standard alternatives, can have advantages over the feeling perceived fit [14,15], enhancing product utility [9], improving aesthetic appeal [3] and better meeting the preferences of customizers [5,14,16]. Furthermore, customization strategy can not only satisfy the aforementioned consumers’ rational demands but also the emotional demands. For instance, consumers can also benefit, as customization provides a sense of accomplishment [5], process enjoyment [14,17], unique need satisfaction [3,18,19], control perception [20] and self-image expression [11].

However, a customization strategy is not always a panacea for success. In addition to the above benefits, customization strategies may also have an adverse impact on purchase intention due to process complexity [9] and decision difficulty [8]. Moreover, in the luxury segment, consumers pay a premium for the designer’s expertise and the status that it can convey [21], and this premium will be positively moderated by consumers’ power distance beliefs [10,22]; however, the product’s expertise signaling value can be eroded by customization. In addition, utilitarian products also reduce consumers’ preference for customization over hedonistic products [18]. From the perspective of consumer characteristics, previous research has found that regret-averse consumers are more reluctant to buy customized products, because they think that the customized ones cannot express what they want [23]. Not only for products, technology-based self-service in complex or high-risk services is also considered a challenge [24].

More importantly, product customization has not only a direct positive or negative effect but also an indirect amplification effect on consumers’ purchase intention. Klesse et al. demonstrates that customization offer consumers an opportunity to extend their self-image [11]. For example, clothes customized or designed by a fashionable customer can also be considered fashionable, and they termed this phenomenon “self-image-consistent product perceptions”. Then, the same is true for food customization and our work believes that consumers also extend their own important characteristics to the focal attributes of customized foods, which can also explain why the preference for customized food varies in different consumers.

### 2.2. The Taste Perception of Customized Foods

Customized foods, as a kind of special food, takes more time and energy for consumers, so they will have a stronger sense of control [20]. Meanwhile, prior research suggests that individuals can convey their self-image onto products over which they have control [25]. Accordingly, consumers will transfer their self-image onto customized foods. In addition, for customized foods consumers usually consider that it will convey diagnostic signals about personal characteristics [26]. In other words, the self-image of consumers can be assessed by their customized foods [27]. Then, consumers will also view the product as an extension of themselves [28] and evaluate it on their own characteristics [29]. Therefore, consumers’ perception or evaluation of customized foods may be influenced by consumers’ own relevant characteristics. Prior research has shown that customization prompted lower healthiness perceptions for individuals who view themselves as unhealthy eaters. Besides healthiness perceptions, taste perception is one of the factors making a difference to consumers’ purchase intention for food, and is even the main factor [12].

Taste perception, which refers to consumers’ perception of whether food tastes in line with their own preferences or flavors [30,31], is also influenced by the producers’ relevant characteristics. Firm or consumer expertise in food is the characteristic related to taste perception. Consumer expertise in food is defined as a fundamental knowledge of food ingredients, higher understanding of options and their tastes and preferences [32,33,34,35], and rises with the increase of food-related experience [36]. Consumer expertise in food plays a crucial role in customizing food [9]. Therefore, we speculate that consumers will incorporate their expertise in the taste perception of the food when customizing food. Furthermore, individuals who consider their expertise higher than that of the merchants will think customized foods tastier, thus are more willing to purchase it. On the contrary, individuals who consider their expertise lower than that of the merchants will think standard foods tastier, and then have a higher purchase intention. Formally, we hypothesize the following:

**Hypothesis** **1.** *Food customization and consumer expertise have an interaction effect on purchase. Specifically, consumers with higher expertise prefer customized food, whereas consumers with lower expertise prefer standard food.*

**Hypothesis** **2.** *Taste perception mediates the interaction effect between food customization and expertise on consumers’ purchase intention.*

**Figure 1:**
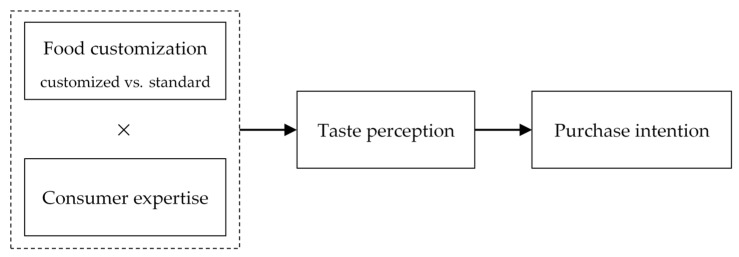
Conceptual model.

Figure 1 depicts our full conceptual model.

## 3. Methodology

We test our proposed framework in two experimental studies carried out in the lab and online separately using a couple of stimulus and product contexts. Study 1 establishes the interaction effect, namely that consumers with higher expertise are more willing to purchase customized foods rather than standard foods. Studies 2 explores the underlying psychological processes of taste perception.

### 3.1. Study 1: The Purchase Intention of Customized Foods

The objective of Study 1 is to directly test H1 by manipulating the customization of an otherwise identical food. We used Subway’s customized sandwich as a prototype for the food customization strategy as a stimulus, providing initial evidence for the interaction effect. We predict that consumers with higher expertise more willing purchase customized foods rather than standard foods.

#### 3.1.1. Method

Participants and design. According to previous research, convenience sampling was used in this study, and two hundred participants (57.50% female; mean age = 33.51 years) were recruited from Sojump panelist (the largest data collection platform in China). Two hundred subjects were randomly assigned to a customizer (N = 100) or non-customizer (N = 100) condition. Study 1 employed a 2 (customization: customized vs. standard) × measured consumer expertise between-subjects design. Participants first completed an attention check that automatically excluded those who failed.

Materials and procedure. Inspired by customized foods stimuli used in the real world and following procedures from customization research [11], we set the stimulus material as a sandwich. We chose sandwiches as the food category for several reasons. First, sandwiches are a popular custom food. Second, it allows addition and removal of ingredients [37]. First, all participants were shown a picture and a brief description of a sandwich. Then participants were randomly assigned to two groups. In the customized foods condition, customizers selected their own ingredients sequentially: one bread (honey oat or cheese), one meat (ham, bacon, or chicken cutlet), one vegetable (cucumber, tomato, or lettuce), and one sauce (salad dressing, mayonnaise, or ketchup). In the standard foods condition, participants received one version of a sandwich (which was either honey oat-ham-cucumber-salad dressing, cheese-bacon-tomato-mayonnaise, or cheese-chicken cutlet-lettuce-ketchup). Within each ingredient category, the ingredients were equal on perceived taste based on a pretest (see Appendix A). Next, participants indicated their purchase intention and consumer expertise on a 7-point Likert scale. We used two items to measure purchase intention (e.g., “I am likely to purchase this sandwich”; 1 = “strongly disagree”, and 7 = “strongly agree”; α = 0.774) [38]. Consumer expertise was measured using the seven items (e.g., “Compared to the average person, I do not know much about sandwiches”, “I am very familiar with sandwiches”, “I am very interested in sandwiches”; 1 = “strongly disagree” and 7 = “strongly agree”; α = 0.934) [39]. Finally, demographic information was reported (for details on our study 1, see Appendix B).

#### 3.1.2. Results and Discussion

Descriptive statistical analysis of the sample. The results of the description and summary statistics of the variables are presented in Table 1. The sample comprised 200 Chinese adults from Sojump. A total of 58.0% of the participants self-reported their love for sandwiches as like or very like; 54.5% of the participants said their frequency of eating sandwiches is at a high level or very high level.

Purchase intention. We conducted a regression analysis with food customization (customized vs. standard), consumer expertise (continuous measure), and their interaction as predictors (Model 1, Hayes 2013). The model revealed a main effect for food customization (B = −2.565, SE = 0.555; t = −4.617, *p* < 0.001, CI_95_ = [−3.660, −1.469]). However, importantly, the interaction hypothesized in H1 was significant (B = 0.643, SE = 0.125; t = 5.165, *p* < 0.001, CI_95_ = [0.398, 0.889]).

**Figure 2:**
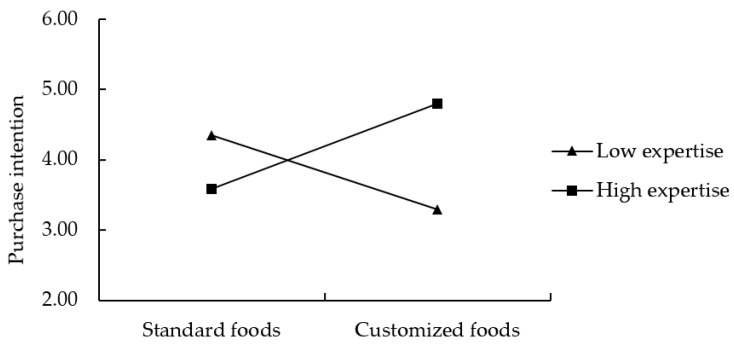
Interaction effect between food customization and consumer expertise in Study 1.

Further spotlight analyses on consumer expertise (M = 4.099, SD = 1.759) illustrated that among those with high consumer expertise (+1SD = 5.858), the purchase intention for yogurt in the standard foods condition (M = 3.590; *p* = 0.001) was lower than that in the customized foods condition (M = 4.793). In contrast, among participants with low consumer expertise (−1SD = 2.341), the purchase intention for yogurt in the standard foods condition (M = 4.355; *p* < 0.001) was higher than that in the customized foods condition (M = 3.295). Thus, H1 was supported (see Figure 2).

In conclusion, Study 1 manipulated the customization strategy by using sandwiches as stimulus material and proved the interaction effect between food customization and expertise on consumers’ purchase intention. In other words, consumers with higher expertise prefer customized foods to standard foods, whereas lower-expertise consumers prefer standard foods.

### 3.2. Study 2: The Mediating Role of Taste Perception

The primary purpose of Study 2 is to test H2 by exploring the underlying psychological processes of taste perception. We directly manipulated consumer expertise and tested a different food customization stimulus to enhance the generalizability of the support for H1. We predict that consumers with lower (higher) expertise are less (more) willing to purchase customized foods due to the lower (higher) taste perception.

#### 3.2.1. Method

Participants and design. Convenience sampling was used in this study, and three hundred and thirty students (51.80% female; mean age = 20.60 years) recruited from Huazhong Agricultural University came into the university lab to participate. Study 2 employed a 2 (customization: customized vs. standard) × 2 (expertise: low vs. high) between-subjects design.

Materials and procedure. We followed procedures from customization research and selected yogurt as a different food customization stimulus because it is quite common for students to customize yogurt [11]. Participants read a short introduction about a new yogurt company. They were told that the researchers had agreed to conduct a market study for this company to assess students’ preferences for yogurts. Participants were individually led into a room and randomly assigned them to a different condition. We use a short passage to manipulate the level of consumer expertise. In the low consumer expertise, we informed participants that “Making yogurt is a difficult task for most consumers and requires mass of specialized knowledge about selecting ingredients”. In the high consumer expertise, participants read “Making yogurt is a simple task for most consumers and only requires a little specialized knowledge about selecting ingredients”. Next, we carried out food customization manipulation. In the customized foods condition, research assistants said that participants can customize their yogurt by selecting four items out of six different options (i.e., apple, banana, pear, walnuts, honey and chocolate chips). They could choose the same ingredient several times. In the standard foods condition, participants received yogurt with certain ingredients. Importantly, a yoked design was adopted to ensure that each participant in the standard condition was assigned an identical yogurt to a participant in the customized condition [40]. Therefore, the overall taste of the yogurt was held constant across conditions.

Then, they filled out a short survey measuring related variables and information (for details, see Appendix C). As a manipulation check, participants were asked about the item “How much expertise do you think you have in yogurt” (1 = “very little”, and 7 = “very much”). Afterwards, participants indicated their purchase intention and perceived taste on a 7-point Likert scale. We measured purchase intention (α = 0.737) using the same items as in Study 1. Taste perception was measured using the four items (e.g., “This yogurt will be delicious”, “This yogurt will be tasty”; 1 = “strongly disagree”, and 7 = “strongly agree”; α = 0.809) [12]. Finally, we asked participants to provide demographic information.

#### 3.2.2. Results and Discussion

Descriptive statistical analysis of the sample. Table 2 presents the description and summary statistics of the variables used in the analysis. The sample comprised 330 students from Huazhong Agricultural University. More than 60% of the participants self-reported their love for yogurt as like or very like. A total of 31.5% of the participants said their frequency of eating yogurt is at an average level.

Manipulation check. ANOVA indicated that our consumer expertise manipulation was successful. As expected, in the high consumer expertise condition, consumers believed that they had higher expertise in yogurt than in the low consumer expertise condition (M_high expertise_ = 4.182, SD_high expertise_ = 1.586, vs. M_low expertise_ = 3.613, SD_low expertise_ = 1.574, *F* = 10.715, *p* = 0.001).

**Figure 3:**
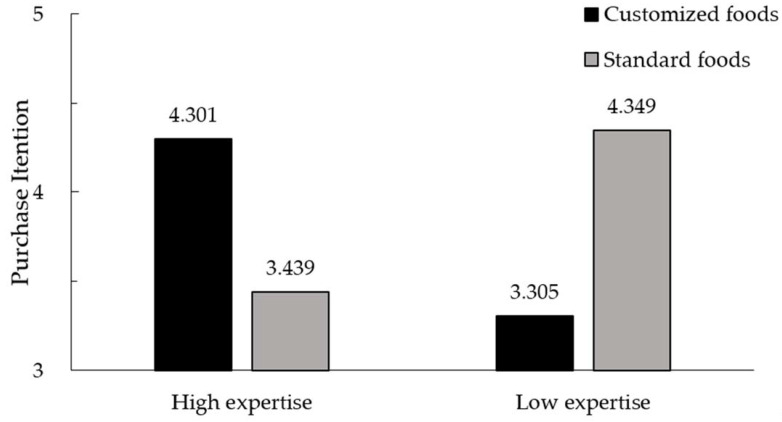
Interaction effect between food customization and consumer expertise in Study 2.

Purchase intention. We conducted 2 × 2 ANOVA on purchase intention with food customization (customized vs. standard) and consumer expertise (low vs. high) as two factors. The model revealed no main effect of food customization (*F* = 0.436, *p* = 0.509). We observed the hypothesized food customization × consumer expertise interaction (*F* = 47.811, *p* < 0.001). Further simple effect analysis illustrated that participants in the high consumer expertise condition were more willing to purchase customized yogurt (M_customized_ = 4.301, SD_customized_ = 1.272) rather than standard yogurt (M_standard_ = 3.439, SD_standard_ = 1.256; *F*(1, 326) = 20.170, *p* < 0.001). In the low consumer expertise condition, participants were more willing to purchase standard yogurt (M_standard_ = 4.349, SD_standard_ = 1.275) rather than customized yogurt (M_customized_ = 3.305, SD_customized_ = 1.193; *F*(1, 326) = 27.845, *p* < 0.001). Similar to the results in Study 1, consumers’ expertise positively affects their preference for customized food. Thus, H1 was supported again (see Figure 3).

Moderated mediation. We ran a moderated mediation analysis to test whether our observed pattern of results between the food customization and consumer expertise on the purchase intention could be explained by variations in perceived taste (PROCESS Model 7) [41]. An index of moderated mediation was significant for identification (index = 1.614; SE = 0. 212, CI_95_ = [1.206, 2.036]). Furthermore, among participants with high consumer expertise, taste perception mediated a positive effect for food customization on purchase intention (B = 0.643, SE = 0.146, CI_95_ = [0.356, 0.932]). In contrast, among participants with low consumer expertise, taste perception mediated a negative effect for food customization on purchase intention (B = −0.971, SE = 0.144, CI_95_ = [−1.257, −0.689]). Thus, these results supported H2.

By directly manipulating consumer expertise, Study 2 tested a different food type (yogurt) to enhance the generalizability of the research conclusion. Consumers with higher expertise preferred customized yogurt to standard yogurt, and consumers with lower expertise preferred standard yogurt to customized yogurt. Importantly, Study 2 not only verified the interaction effect in Experiment 1 again, but also further showed that this effect is mediated by taste perception.

## 4. General Discussion

### 4.1. Conclusions

Given a large body of studies have shown that evidence is mixed on consumer preference for customized foods, in our research we focused on the food field to examine the interaction effect between food customization and consumer expertise on purchase intention and taste perception as the underlying mechanism through two experiments (sandwich and yogurt). Study 1 suggests that the effect of food customization on consumers’ purchase intention is moderated by their expertise. Specifically, consumers with higher expertise would rather buy customized foods than the standard foods recommended by the merchants. Consumers with lower expertise are more willing to purchase standard foods rather than customized foods. Study 2 further demonstrates the mediating role of taste perception. Taste perception mediates the interaction effect between food customization and expertise on consumers’ purchase intention.

### 4.2. Theoretical Implications

We make several theoretical contributions to the literature. Our work contributes to research on customization by exploring consumer preference for food customization in the food field. Whereas prior work has probed into consumer preference for food customization, most of it focuses on the direct effect of customization, especially the positive effect, such as enhancing product utility [9], improving aesthetic appeal [3], process enjoyment [14,17], unique need satisfaction [3,18], control perception [20] and self-image expression [11]. Other research explored the negative effect of food customization, such as process complexity [9] and decision difficulty [8]. However, customization also has an important indirect effect. Klesse et al. posited that customized products can be viewed as an extension of a customizers’ self-image and they term this conclusion “self-image-consistent product perceptions” [11]. Further focusing on customized food, our work suggests that food customization prompts customers to perceive focal food attributes in line with their relevant prominent characteristics.

Second, we explicate a psychological mechanism for taste perception that explains consumers’ preference for standard foods. Previous research suggests there are multiple motivations underlying consumers’ preference for customized versus standard foods, such as a sense of accomplishment [5], a sense of engagement [4] and unique need satisfaction [18]. However, taste perception is undoubtedly the most apparent factor affecting purchase intention for customized foods. Therefore, our work elucidates the general feeling that our work improves the extent of understanding of consumers’ preference for food customization by revealing the mediating role of taste perception.

Finally, we identify consumer expertise as a moderator of reactions to customized versus standard foods and thereby uncover a segmentation variable that rests within the consumer. Consumer expertise as a significant characteristic related to taste perception, plays a crucial role in customizing food [9]. Consumers consider their expertise as an evaluation criterion for the taste perception of customized foods. Specifically, low-expertise consumers will perceive the standard foods (vs. customized foods) tastier, thus they are more reluctant to buy customized foods. In contrast, for consumers with high expertise, they may believe customized foods tastier and are more willing to purchase them.

### 4.3. Managerial Implications

Our findings also have some important implications for marketing managers that rely on customization as part of their business strategy. We urge marketing managers to proceed cautiously when implementing food customization initiatives, because consumers with different levels of expertise have diverse preferences for customized foods. For regular customers or those who know more about a food, marketing managers can encourage them to customize the food. However, for novice consumers or those who know little about a food, standard foods should be recommend to them to increase their purchase intention. Furthermore, consumer expertise is low when a new kind of food is introduced, so marketing managers should avoid adopting the strategy of food customization to prevent the adverse effect. Conversely, marketing managers should take effective measures to improve consumer expertise when adopting the strategy of food customization. For instance, the provision of guidance during the customization process will attenuate this negative effect of food customization.

### 4.4. Limitations and Future Research

Our results are consistent but not without several limitations. First, we only conducted two empirical studies without real field data to provide further support for our theory, which may weaken the external validity of the research. Future research can explore the effect of food customization through the real data of a company. Second, only two types of food were used in this study, sandwich and yogurt, and future research can explore the effect of food customization for different food types. For example, for functional food, consumers tend to pay more attention to its utilitarian value and ignore the taste perception, so as to change the effect of food customization. Finally, our studies have revealed the underlying mechanism of the effect of food customization on purchase intention–taste perception, there may be other mechanisms that we have overlooked to explain this effect and deserve further exploration.

## Figures and Tables

**Table 1:** Definition and summary statistics of selected variables of Study 1.

Variable	Description	Mean	SD
Age	Age of participant	33.51	8.756
Gender	Gender of participant (1 if male, 0 otherwise)	0.43	0.496
Education	Participant’s maximal education level (1= Junior high school and below; 2 = senior high school or technical secondary school; 3 = junior college; 4 = undergraduate; 5 = Postgraduate and above)	3.04	1.065
Income	Average monthly income of participant (1 = below 3000 yuan; 2 = 3000~6000 yuan; 3 = 6000~10,000 yuan; 4 = 10,000~15,000 yuan; 5 = 15,000 yuan and above)	2.96	1.107
Love	How much you love sandwiches? (1 = very little; 5 = very much)	3.56	1.176
Frequency	How often do you have sandwiches? (1 = very little; 5 = very frequent)	3.42	1.350

SD: Standard deviation.

**Table 2:** Definition and summary statistics of selected variables of Study 2.

Variable	Description	Mean	SD
Age	Age of participant	20.60	1.891
Gender	Gender of participant (1 if male, 0 otherwise)	0.52	0.500
Income	Family incomes per capita (1 = below 1000 yuan; 2 = 1000~3000 yuan; 3 = 3000~6000 yuan; 4= 6000~10,000 yuan; 5 = 10,000 yuan and above)	2.98	1.013
Love	How much you love yogurt? (1 = very little; 5 = very much)	3.68	1.267
Frequency	How often do you have yogurt? (1 = very little; 5 = very frequent)	3.08	1.140

## Data Availability

The data presented in this study are available on request from the corresponding author.

## Appendix A

**Table A1:** Pre-test and selected ingredients.

Categories	All Ingredients Included in the Pre-Test	Selected Ingredients and Perceived Taste
Breads	honey oat bread	Honey oat bread; M_taste_ = 6.42 Cheese bread; M_taste_ = 6.49
	cheese bread	
	white bread	
	wholemeal bread	
Meats	ham	Ham; M_taste_ = 7.94 Bacon; M_taste_ = 8.06 Chicken cutlet; M_taste_ = 8.11
	bacon	
	chicken cutlet	
	beef	
	shrimp	
	tuna	
Vegetables	cucumber	Tomato; M_taste_ = 5.28 Cucumber; M_taste_ = 5.16 Lettuce; M_taste_ = 5.26
	tomato	
	lettuce	
	green pepper	
	onion	
	carrot	
	sweet potato	
Sauces	salad dressing	Salad dressing; M_taste_ = 6.95 Mayonnaise; M_taste_ = 6.84 Ketchup; M_taste_ = 6.88
	mayonnaise	
	chili sauce	
	barbecue sauce	
	ketchup	

*N* = 109 (56.90% female; mean age = 33.87, SD = 8.93). Taste measured on an 11-point Likert scale (0 = not tasty at all to 10 = extremely tasty).

## Appendix B

### Study 1 and the Whole Questionnaire

#### Dear Interviewee:

We are the Food Marketing Research Center of Huazhong Agricultural University. In order to understand the attitudes of consumers towards fast food, the research center specially compiled this questionnaire. This questionnaire adopts an anonymous survey method, and we hope you can actively cooperate and fill in your real situation. This questionnaire will be kept confidential and used for academic research only.

**First, please browse the following text and pictures carefully**

A sandwich is a typical Western food. It is made of two pieces of bread with several slices of meat, some vegetables and various spices.

- (Customized situation)

Imagine that you are buying a sandwich at a fast-food restaurant, you are told to select your own ingredients.

First, you need to choose one of two types of bread, which one would you choose?

□ Honey oat

□ Cheese

Second, you need to choose one of three types of meat, which one would you choose?

□ Ham

□ Bacon

□ Chicken cutlet

Third, you need to choose one of three types of vegetable, which one would you choose?

□ Cucumber

□ Tomato

□ Lettuce

Last, you need to choose one of three types of sauce, which one would you choose?

□ Salad dressing

□ Mayonnaise

□ Ketchup

- (Standard situation)

Imagine that you are buying a sandwich at a fast-food restaurant, you are told that the sandwich you can buy right now is honey oat-ham-cucumber-salad dressing/cheese-bacon-tomato-mayonnaise/cheese-chicken cutlet-lettuce-ketchup.

**Please answer the following questions based on the situation**

I am likely to purchase this sandwich.

strongly disagree □ 1 □ 2 □ 3 □ 4 □ 5 □ 6 □ 7 strongly agree

I am going to buy the sandwich.

strongly disagree □ 1 □ 2 □ 3 □ 4 □ 5 □ 6 □ 7 strongly agree

**Please answer the following questions**

Compared to the average person, I do not know much about sandwiches. (reverse).

strongly disagree □ 1 □ 2 □ 3 □ 4 □ 5 □ 6 □ 7 strongly agree

I am very familiar with sandwiches.

strongly disagree □ 1 □ 2 □ 3 □ 4 □ 5 □ 6 □ 7 strongly agree

I am not knowledgeable about sandwiches. (reverse)

strongly disagree □ 1 □ 2 □ 3 □ 4 □ 5 □ 6 □ 7 strongly agree

I am very interested in sandwiches.

strongly disagree □ 1 □ 2 □ 3 □ 4 □ 5 □ 6 □ 7 strongly agree

I buy sandwiches a lot.

strongly disagree □ 1 □ 2 □ 3 □ 4 □ 5 □ 6 □ 7 strongly agree

My friends buy sandwiches a lot.

strongly disagree □ 1 □ 2 □ 3 □ 4 □ 5 □ 6 □ 7 strongly agree

I read about sandwiches (e.g., reviews, blogs, inserts, ads, flyers) all the time.

strongly disagree □ 1 □ 2 □ 3 □ 4 □ 5 □ 6 □ 7 strongly agree

**Please fill in the following questions truthfully**

Your gender:

□ Male

□ Female

Your age: __________

Your maximal education level is:

□ Junior high school and below

□ Senior high school or technical secondary school

□ Junior college

□ Undergraduate

□ Postgraduate and above

Your average monthly income:

□ Below 3000 yuan

□ 3000~6000 yuan

□ 6000~10,000 yuan

□ 10,000~15,000 yuan

□ 15,000 yuan and above

How much you love sandwiches?

□ Very little

□ Little

□ Medium

□ Much

□ Very much

How often do you have sandwiches?

□ Very little

□ Little

□ Medium

□ Frequent

□ Very frequent

## Appendix C

### Study 2 and the Short Survey

**Please fill in the following questions**

How much expertise do you think you have in yogurt?

very little □ 1 □ 2 □ 3 □ 4 □ 5 □ 6 □ 7 very much

**Please fill in the following questions**

I am likely to purchase this yogurt.

strongly disagree □ 1 □ 2 □ 3 □ 4 □ 5 □ 6 □ 7 strongly agree

I am going to buy the yogurt.

strongly disagree □ 1 □ 2 □ 3 □ 4 □ 5 □ 6 □ 7 strongly agree

**Please fill in the following questions**

strongly disagree □ 1 □ 2 □ 3 □ 4 □ 5 □ 6 □ 7 strongly agree

This yogurt will be tasty.

strongly disagree □ 1 □ 2 □ 3 □ 4 □ 5 □ 6 □ 7 strongly agree

This yogurt will be flavorful.

strongly disagree □ 1 □ 2 □ 3 □ 4 □ 5 □ 6 □ 7 strongly agree

This yogurt will be palatable.

strongly disagree □ 1 □ 2 □ 3 □ 4 □ 5 □ 6 □ 7 strongly agree

**Please fill in the following questions truthfully**

Your gender:

□ Male

□ Female

Your age: __________

Your family incomes per capita:

□ Below 1000 yuan

□ 1000~3000 yuan

□ 3000~6000 yuan

□ 6000~10,000 yuan

□ 15,000 yuan and above

How much you love yogurt?

□ Very little

□ Little

□ Medium

□ Much

□ Very much

How often do you have yogurt?

□ Very little

□ Little

□ Medium

□ Frequent

□ Very frequent
